# The psychosocial functioning in adolescents with severe obesity evaluated throughout the strengths and difficulties questionnaire (SDQ): a preliminary report

**DOI:** 10.3389/fpsyg.2023.1205113

**Published:** 2024-01-15

**Authors:** Anna Guerrini Usubini, Michela Bottacchi, Giovanna Morelli, Diana Caroli, Nicoletta Marazzi, Gianluca Castelnuovo, Alessandro Sartorio

**Affiliations:** ^1^Psychology Research Laboratory, Istituto Auxologico Italiano, Istituto di Ricovero e Cura a Carattere Scientifico (IRCCS), Milan, Italy; ^2^Division of Auxology, Istituto Auxologico Italiano, Istituto di Ricovero e Cura a Carattere Scientifico (IRCCS), Piancavallo-Verbania, Italy; ^3^Sacra Famiglia Foundation, Pediatric Neuropsychiatry Service, Cocquio Trevisago-Varese, Italy; ^4^Experimental Laboratory for Auxo-Endocrinological Research, Istituto Auxologico Italiano, Istituto di Ricovero e Cura a Carattere Scientifico (IRCCS), Piancavallo-Verbania, Italy; ^5^Experimental Laboratory for Auxo-Endocrinological Research, Istituto Auxologico Italiano, Istituto di Ricovero e Cura a Carattere Scientifico (IRCCS), Milan, Italy; ^6^Department of Psychology, Catholic University of Milan, Milan, Italy

**Keywords:** childhood obesity, adolescents, psychological adjustment, psychological wellbeing, strengths and difficulties questionnaire

## Abstract

**Introduction:**

Childhood obesity is associated with poor psychological adjustment. Severely impacts the psychological adjustment of young patients. To assess the psychological functioning of children and adolescents, several questionnaires have been proposed so far. Although the Strengths and Difficulties Questionnaire (SDQ) is one of the most well-used tools, its application in obesity research is scarce. The study is aimed at assessing the psychological profile of a sample of Italian children and adolescents seeking an in-hospital multidisciplinary body weight reduction program for obesity, via SDQ.

**Methods:**

One hundred and fourteen consecutive Italian children and adolescents with obesity (43 males/71 females, age range: 11–17 years, mean age ± SD: 15.1 ± 1.66, body mass index-BMI ± SD: 37.4 ± 6.13 kg/m^2^), were recruited at the Division of Auxology, Istituto Auxologico Italiano IRCCS, Piancavallo (VB).

**Results:**

Obese Females reported worse conditions of emotional symptoms (*t* = 5.48; *p* < 0.001) and peer problems (*t* = 2.34; *p* = 0.021), as well as higher which were associated with greater scores of pro-social behaviors than obese males (*t* = 3.07; *p* = 0.003). The total difficulties score (*t* = 4.00; *p* < 0.001) and the total impact score (*t* = 4.53; *p* < 0.001) were significantly higher in females than males. No statistically significant differences in SDQ variables were found in relation to the degree of obesity (BMI SDS: 2–2.99; BMI SDS: > 3).

**Discussion:**

These findings can contribute to understand the psychological condition of adolescents with obesity in a better way and also to develop effective interventions for the treatment of pediatric obesity which not only take into account the medical and physical aspects but also the emotional and social difficulties expressed by adolescents with obesity.

## 1 Introduction

Childhood obesity is one of the major public health challenges of the 21st century (World Health Organization, 2021). In the world, around 42 million children under the age of five are estimated to be overweight or obese, and the prevalence is still increasing. It has been calculated that, by 2025, about 70 million children will be overweight or obese (Ng et al., 2014). Among European countries, Italy shows one of the highest rates of excess weight among school-aged children, with 20.4% of children with overweight and 9.4% with obesity (World Health Organization, 2017). Children with obesity demonstrate an increased risk of remaining obese in adulthood (Simmonds et al., 2016). In addition, childhood obesity is associated with cardiovascular, respiratory, and metabolic diseases, hypertension, cancer and early mortality in adulthood (Weihrauch-Blüher et al., 2019). As far as mental health is concerned, adolescent obesity is associated with many comorbidities (Drosopoulou et al., 2021; Kokka et al., 2023), including depression (Sutaria et al., 2019), anxiety, low self-esteem, poor quality of life, body dissatisfaction, and other emotional and behavioral problems (Griffiths et al., 2011; Rankin et al., 2016). Significant associations were also found between obesity and attention-deficit/hyperactivity disorder (ADHD) (Erhart et al., 2012; Khalife et al., 2014) and conduct problems (Nujić et al., 2021). Additional findings (Marks et al., 2009) showed that obesity is associated with many psychosocial problems such as stigma, teasing, and bullying. Such psychological conditions are pervasive and can have serious consequences for emotional and physical health.

Adolescence is a particularly high-risk time for weight gain due to the naturally occurring metabolic changes of puberty, but obese children have been found to gain more weight than their normal-weight peers (Jasik and Lustig, 2008). In addition, many studies confirm that overweight and obesity in childhood can influence early puberty, mainly in females (Polat, 2022). In a recent study, earlier pubertal timing was associated with several psychological distress and behavioral problems, such as more alcohol, nicotine, and cannabis use, more risky behavior, emotional distress, greater likelihood of being bullied, and more symptoms of major depressive disorder, and adult antisocial behavior (Padrutt et al., 2023).

The psychological functioning of children and adolescents with obesity can be investigated with a range of assessment tools, including the Strengths and Difficulties Questionnaire (SDQ; Goodman, 1997). Although SDQ is one of the most used instruments to assess the psychological adjustment of children and youth only few articles have been published about the psychological profile of children and adolescents with obesity via SDQ (Griffiths and Page, 2008; Beynon, 2023; Donnchadha et al., 2023), and none of them were carried out in the Italian context.

Thus, the current study aims to assess the psychological profile of a sample of Italian adolescents with obesity, via SDQ, addressing their social, emotional, and behavioral functioning. Specifically, any potential differences between genders as well as degrees rates of obesity were assessed.

## 2 Materials and methods

### 2.1 Participants and procedures

Participants of the current cross-sectional study were one hundred and nineteen consecutive Italian adolescents with obesity (46 males, 73 females), mean age 15.1 (SD = 1.66), mean body mass index (BMI ± SD: 37.4 ± 6.13 kg/m^2^). Participants were recruited at the Division of Auxology, Istituto Auxologico Italiano IRCCS, Piancavallo (VB), a clinical center for multidisciplinary obesity rehabilitation. Inclusion criteria were: (1) being Italian; (2) aged between 11 and 17 years; and (3) having BMI > 97th centile (Cacciari et al., 2006). Exclusion criteria comprised any form of physical or mental impairment that could compromise participation in the study. After being informed about the research and after obtaining both written informed consent to participate from their parents and assent from the young patients, participants were screened for participation in the study. A clinical interview was conducted by an independent psychologist with expertise in clinical psychology to assess inclusion/exclusion criteria. All patients who were asked to participate met all the inclusion criteria. Once enrolled, participants were asked to provide socio-demographic data and fill in self-report questionnaires at the beginning of a 3-week body weight reduction program. A total of 120 participants were initially enrolled, but one of them did not provide written informed consent to participate. Five subjects (3 males, and 2 females) were excluded due to an incomplete compilation of the questionnaire, thus reducing the study population analyzed in the present study to 114 adolescents with obesity (43 males, 71 females). Data were collected between May and November 2022.

The current study was approved by the Ethical Committee of Istituto Auxologico Italiano, IRCCS, Milan, Italy (approval number: 2021_01_26_03). Research was carried out according to the Declaration of Helsinki and its advancements.

### 2.2 Measures

Socio-demographic data: Respondents were asked to provide information about gender, age, educational level, and socio-economic status. Such information was self-reported.

Anthropometric data: Weight and height were measured by the internal medical staff to calculate BMI according to the proper formula: kg/m^2^. Standing height was determined by a Harpenden Stadiometer (Holtain Limited, Crymych, Dyfed, UK). Weight was measured to the nearest 0.1 kg using an electronic scale (RoWU 150, Wunder Sa.bi., Trezzo sull’Adda, Italy).

Psychological adjustment: The Strengths and Difficulties Questionnaire (Goodman, 1997) Italian validation (Di Riso et al., 2010) was used as a screening tool for assessing psychological adjustment in children and adolescents. It is a valid and well-suitable 25 items questionnaire that has been designed to assess emotional and behavioral problems in young subjects. The SDQ has four subscales to measure major difficulties commonly experienced by children and adolescents (conduct problems, hyperactivity inattention, emotional symptoms, peer problems) and one subscale to assess strengths (pro-social behavior). Each subscale of the SDQ contains five items which are rated on a three-point Likert-type scale (0 = not true, 1 = somewhat true, or 2 = certainly true). Each subscale is calculated by adding scores on the relevant items (after reversing indicated items). All but the last are summed to generate a total difficulties score, with higher scores reflecting greater difficulties. Higher scores on the pro-social behavior subscale reflect more strength. Five additional items that investigate how difficulties upset or distress the child and interfere with home life, friendship, classroom learning, and leisure activities are summarized to produce a total impact score. Since its first publication in 1997, the SDQ has been translated into more than 40 languages and it has been widely and successfully adopted in both clinical and research settings (Theunissen et al., 2019).

Demographical and clinical data were collected via self-report.

### 2.3 Statistical analysis

All analyses were performed with Jamovi (The jamovi project 2021). jamovi (Version 1.6) [Computer Software] retrieved from https://www.jamovi.org.

Descriptive statistics were computed for all demographical, physical, and clinical variables. Categorical variables were presented in frequencies and percentages, continuous variables were expressed in means and standard deviations. The normal distribution of the variables was assessed by skewness and kurtosis indices. Relations between all the SDQ subscales were assessed with Pearson’s correlation. To assess gender differences as well as differences in different degrees rates of obesity in all the SDQ variables independent samples *t*-tests were run.

Initially, analysis was performed in the total sample, then, we explored the difference between males and females and finally between different degrees of obesity according to the BMI standard deviation score (BMI SDS) (Group 1: BMI SDS 2-2.99 vs. Group 2: BMI SDS > 3).

Group 1 (*N* = 61) was composed of 23 males and 38 females, mean age of 15.5. Group 2 (*N* = 53) was composed of 20 males and 33 females, mean age of 15.7.

Critical alpha was set to 0.05. Cohen’s *d* was used to quantify effect size.

## 3 Results

The sample was composed of 114 consecutive Italian adolescents with obesity (43 males, 71 females), aged between 11 and 17, mean age 15.1 (SD = 1.66), mean body mass index (BMI ± SD: 37.4 ± 6.13 kg/m^2^), seeking a 3-week period of hospitalization for a multidisciplinary body weight reduction program. Most of the sample completed middle school (80%), lived in the Northern regions of Italy (74%) in families with low-to-median socio-economic status (68%).

We analyzed the scores of SDQ from a qualitative and then a quantitative point of view.

### 3.1 Qualitative interpretation of SDQ

Each subscale and total SDQ score can be qualitatively interpreted as “normal,” “abnormal” or “borderline” according to the original categorization (Di Riso et al., 2010).

Assuming this qualitative point of view, in the total sample, 35.1% of participants showed abnormal mean scores of emotional symptoms, 16.7% showed abnormal scores of conduct problems, and 18.4% had abnormal scores of hyperactivity-inattention. Peer problems emerged as an abnormal score in 26.3% of participants, and pro-social behaviors were abnormal in 1.8% of participants. The total difficulties score was abnormal in 27.2% of subjects and the total impact score was abnormal in 52.6% of the sample, suggesting a significant general negative impact of psychological difficulties in their life domains. Frequencies and percentages of normal, borderline, and abnormal scores of SDQ of the total sample are presented in Table 1. For each subscale, range scores for the three each categories (normal, abnormal, and borderline) are presented in Table 2.

**TABLE 1:** Frequencies and percentages of different scores of the single subjects in SDQ subscales.

SDQ subscales		Range		
		Normal (*N* %)	Borderline (*N* %)	Abnormal (*N* %)
Emotional symptoms	F	28 (39.44)	8 (11.27)	35 (49.29)
	M	35 (81.40)	3 (6.98)	5 (11.63)
	Group 1	37 (60.66)	5 (8.20)	19 (31.15)
	Group 2	26 (49.06)	6 (11.32)	21 (39.62)
Conduct problems	F	50 (70.42)	7 (9.86)	14 (19.72)
	M	35 (81.40)	3 (6.98)	5 (11.63)
	Group 1	42 (68.85)	6 (9.84)	13 (21.31)
	Group 2	43 (81.13)	4 (7.55)	6 (11.32)
Hyperactivity-inattention	F	43 (60.56)	12 (16.90)	16 (22.54)
	M	33 (76.74)	5 (11.63)	5 (11.63)
	Group 1	44 (72.13)	7 (11.48)	10 (16.39)
	Group 2	32 (60.38)	10 (18.87)	11 (20.75)
Peer problems	F	28 (39.44)	22 (30.99)	21 (29.56)
	M	28 (65.11)	6 (13.95)	9 (20.93)
	Group 1	29 (47.54)	16 (26.23)	16 (26.23)
	Group 2	27 (50.94)	12 (22.64)	14 (26.41)
Pro-social behaviors	F	66 (92.96)	3 (4.23)	2 (2.82)
	M	37 (86.05)	6 (13.95)	0
	Group 1	55 (90.16)	4 (6.56)	2 (3.28)
	Group 2	48 (77.05)	5 (9.43)	0
Total difficulties	F	26 (36.62)	19 (26.76)	26 (36.62)
	M	30 (69.77)	8 (18.60)	5 (11.63)
	Group 1	31 (50.82)	14 (22.95)	16 (26.23)
	Group 2	25 (47.17)	13 (24.53)	5 (9.43)
Total impact	F	14 (19.72)	7 (9.86)	50 (70.42)
	M	28 (65.12)	5 (11.63)	10 (23.26)
	Group 1	21 (34.43)	8 (13.11)	32 (52.46)
	Group 2	21 (39.62)	4 (7.55)	28 (52.83)

F, females; M, males; group 1, BMI SDS 2-2.99; group 2, BMI SDS > 3.

**TABLE 2:** Means and standard deviations.

SDQ subscales		M (SD)	Range		
			Normal	Borderline	Abnormal
Emotional symptoms	F	5.83 (2.59)	0–5	6	7–10
	M	3.12 (2.52)			
	Group 1	4.74 (2.89)			
	Group 2	4.89 (2.89)			
Conduct problems	F	2.89 (1.82)	0–3	4	5–10
	M	2.26 (1.68)			
	Group 1	2.82 (1.87)			
	Group 2	2.45 (1.69)			
Hyperactivity-inattention	F	4.66 (2.22)	0–5	6	7–10
	M	3.93 (2.04)			
	Group 1	4.31 (2.00)			
	Group 2	4.47 (2.37)			
Peer problems	F	4.10 (2.30)	0–3	4–5	6–10
	M	3.05 (2.38)			
	Group 1	3.79 (2.30)			
	Group 2	3.60 (2.48)			
Pro-social behavior	F	8.48 (1.53)	6–10	5	0–4
	M	7.56 (1.59)			
	Group 1	8.03 (1.68)			
	Group 2	8.25 (1.53)			
Total difficulties score	F	17.34 (6.47)	0–15	16–19	20–40
	M	12.35 (6.44)			
	Group 1	15.66 (6.74)			
	Group 2	15.23 (7.09)			
Total impact score	F	3.30 (2.85)	0	1	2–10
	M	1.07 (1.94)			
	Group 1	2.36 (6.67)			
	Group 2	2.57 (2.87)			

F, females; M, males; group 1, BMI: SDS 2-2.99; group 2, BMI: SDS > 3.

Females reported having borderline mean scores of peer problems. Borderline scores were also found in females as well as both groups 1 and 2 as far as emotional symptoms, total difficulties scores, and total impact scores are concerned. Means and standard deviations in all the subscales of SDQ divided by gender and subgroups are shown in Table 2.

### 3.2 Quantitative interpretation of SDQ

When comparing males and females in all the SDQ variables, girls reported worse conditions of emotional symptoms (*t* = 5.48; *p* < 0.001) with a large effect size (Cohen’s *d* = 1.059) and peer problems (*t* = 2.34; *p* = 0.021) with a small-to-medium effect size (Cohen’s *d* = 0.451) as well as higher greater scores of pro-social behaviors than boys (*t* = 3.07; *p* = 0.003) with a medium effect size (Cohen’s *d* = 0.593). The total difficulties score (*t* = 4.00; *p* < 0.001) and the total impact score (*t* = 4.53; *p* < 0.001) both with large effect sizes (Cohen’s *d* = 0.772 and Cohen’s *d* = 0.875, respectively) were significantly higher in females than males. No other significant differences in SDQ between females and males were found. Comparisons between males and females in SDQ are reported in Table 3.

**TABLE 3:** Comparisons between males and females in SDQ.

	Student’s t	*p*	Mean differences	SE differences	Cohen’s d effect size
Emotional symptoms	5.48	<0.001	2.715	0.495	1.059
Conduct problems	1.85	0.068	0.632	0.342	0.357
Hyperactivity-inattention	1.76	0.082	0.732	0.417	0.339
Peer problems	2.34	0.021	1.052	0.450	0.451
Pro-social behaviors	3.07	0.003	0.921	0.300	0.593
Total difficulties	4.00	<0.001	4.989	1.249	0.772
Total impact	4.53	<0.001	2.226	0.492	0.875

As far as differences in relation to different degrees of obesity is concerned, no statistically significant differences between groups 1 and 2 in SDQ variables were found (see Table 4).

**TABLE 4:** Comparison between group 1 (BMI SDS 2-2.99) and group 2 (BMI SDS > 3) in SDQ.

	Student’s t	*p*	Mean differences	SE differences	Cohen’s d effect size
Emotional symptoms	−0.275	0.784	2.715	0.495	−0.0517
Conduct problems	1.092	0.277	0.632	0.342	0.2051
Hyperactivity-inattention	−0.391	0.697	0.732	0.417	−0.0734
Peer problems	0.409	0.683	1.052	0.450	0.0768
Pro-social behaviors	−0.701	0.485	0.921	0.300	−0.1317
Total difficulties	0.331	0.741	4.989	1.249	0.0622
Total impact	−0.395	0.693	2.226	0.492	−0.0742

SDS, standard deviation score.

Correlations between all the SDQ subscales showed that emotional symptoms, conduct problems, hyperactivity-inattention, and peer problems were positively and significantly related to each other (*p* < 0.001 for all the correlations). Pro-social behavior was negatively associated with peer problems suggesting that the higher the level of pro-social behavior, the lower the level of peer problems. No other significant correlations were found. Correlations are presented in Table 5.

**TABLE 5:** Correlations between SDQ subscales.

	Emotional symptoms	Conduct problems	hyperactivity-inattention	Peer problems	Pro-social behaviors	Total difficulties	Total impact
**Emotional symptoms**							
Conduct problems	0.451***	−					
Hyperactivity-inattention	0.424***	0.485***	−				
Peer problems	0.484***	0.302**	0.307***	−			
Pro-social behaviors	-0.029	-0.288**	-0.156	-0.189**	−		
Total difficulties	0.818***	0.718***	0.716***	0.700***	-0.184**	−	
Total impact	0.660***	0.422***	0.499***	0.483***	-0.052	0.685***	−

***p < 0.001;

**p < 0.05.

## 4 Discussion

In this research, we evaluated the psychological profile of a sample of Italian children and adolescents with obesity, seeking an in-hospital multidisciplinary body weight reduction program, via SDQ.

Taking a qualitative perspective, we found several indicators of psychological concerns in children/adolescents with obesity, as shown by an “abnormal” impact score of SDQ, indicating how heavy the impact of difficulties that these subjects experienced in many life domains is. Accordingly, several findings in the literature suggest that the condition of obesity can severely impact the psychological and social adjustment (Griffiths and Page, 2008; Pulgarón, 2013; Erskine et al., 2015; Beltrán-Garrayo et al., 2023). Literature also suggests that the condition of childhood obesity is linked to some psychosocial disadvantages such as parental divorce (Hamer and Stamatakis, 2008; Gundersen et al., 2011) and low family income (Hanson and Chen, 2007).

In light of our results, peer relationships seem to be one of the impaired areas in the mental functioning of Italian adolescents with obesity seeking an in-hospital multidisciplinary body weight reduction program. Young subjects suffering from obesity are often subjects of stigma and victimization. Weight stigma in youth occurs most frequently in the form of teasing and bullying from peers about body weight. For youth, experiences of weight stigma can be particularly damaging, with long-lasting consequences that negatively affect their life course emotional and physical wellbeing (Puhl and Lessard, 2020). In some studies of the literature, girls have been found to experience more weight stigma when compared to boys (Salmon et al., 2018) and related negative consequences on mental health (Juvonen et al., 2019). Signs of low mean scores of emotional wellbeing in females emerged also in our study. SDQ revealed a “borderline” score of total difficulties and emotional symptoms in young girls suggesting a quite clinically relevant impairment in their emotional wellbeing.

Even assuming a quantitative approach and exploring differences between genders in all the dimensions of SDQ, we found consistent results that showed greater levels of emotional symptoms and peer problems as well as total difficulties and the total impact of females than males. Such results are independent of the degree of obesity, as shown by comparable values of SDQ items between the two subgroups (group 1: BMI SDS 2-2.99 vs. group 2: BMI SDS > 3). Consistently with our results, large differences between genders have been detected in this domain across literature, as suggested by numerous studies in which females were more likely to show worse psychological conditions than males, including emotional problems such as depression and anxiety (Phillips et al., 2012).

Our results also showed significantly higher scores in pro-social behaviors in females than in males, this finding being in line with the literature, where girls were found to report greater levels of pro-social behavior than boys (Carlo et al., 2007). It is worth noting that, in our study, negative associations between pro-social behaviors and conduct problems as well as peer problems were found, suggesting that the higher the level of pro-social behavior the lower the level of conduct and peer problems. Such evidence leads us to read pro-social behavior as a potentially protective factor against the onset of problematic psychological functioning but, further investigation in this direction is required.

The current study presents some strengths. It is one of the few attempts to investigate the psychological functioning of children/adolescents with obesity using SDQ in the Italian context. In addition, it has been carried out in a highly specialized clinical center for obesity that allows us to reach a sample of a specific and clinically relevant population of children and adolescents with a high degree of obesity.

However, some limitations of the study need to be pointed out. First, the cross-sectional nature of the study with a lack of comparison with a control group of normal-weight peers and no longitudinal measures prevented us from leading causal conclusions. Second, the use of only a self-report measure for psychological adjustment carried out a risk for biases and so, required caution in the interpretation of the data. Third, the sample was recruited from a single clinical center in Italian adolescents with obesity seeking an in-hospital multidisciplinary body weight reduction program and, thus, could be scarcely representative of the entire population.

## 5 Conclusion

Despite the above limitations, the present study provides evidence for several difficulties in the psychological adjustment in adolescents with obesity. In summary, a high prevalence of “abnormal scores” in the total impact score, emotional problems, and peer problems was found, with less than 50% of female participants being in the normal range.

We also found that higher emotional symptoms and peer problems as well as total difficulties and the total impact scores were reported by females in comparison with males, and peer problems seem to be one of the most impaired area in the mental functioning of obese adolescents. Furthermore, no significant differences were found between the two BMI subgroups.

Such evidence can be useful to understand the psychological condition of adolescents with obesity in a better way, and, concurrently, develop effective interventions for the treatment of pediatric obesity which not only take into account the medical and physical aspects but also the emotional and social difficulties expressed by adolescents with obesity.

Nevertheless, further effort is required to explore the extent and implications of psychological impairment in adolescent obesity to deeply understand the phenomenon as well as to provide efficient interventions. Future directions of the study toward collecting additional data about family and economic conditions of adolescents with obesity should be depicted to deeply understand the impact of psychosocial factors on adolescents with obesity development.

## Data availability statement

The raw data supporting the conclusions of this article will be made available by the authors, without undue reservation.

## Ethics statement

The current study was approved by the Ethical Committee of Istituto Auxologico Italiano, IRCCS, Milan, Italy (approval number: 2021_01_26_03). The studies were conducted in accordance with the local legislation and institutional requirements. Written informed consent for participation in this study was provided by the participants’ legal guardians/next of kin.

## Author contributions

AG and AS: conceptualization. AG: formal analysis and writing—original draft preparation. MB, GM, DC, and NM: data curation. GC and AS: writing—review and editing and supervision. All authors have read and agreed to the published version of the manuscript.
